# Distinct evolutionary strategies in the GGPPS family from plants

**DOI:** 10.3389/fpls.2014.00230

**Published:** 2014-05-27

**Authors:** Diana Coman, Adrian Altenhoff, Stefan Zoller, Wilhelm Gruissem, Eva Vranová

**Affiliations:** ^1^Department of Biology, ETH ZurichZurich, Switzerland; ^2^Department of Computer Science, ETH ZurichZurich, Switzerland; ^3^Swiss Institute of BioinformaticsZurich, Switzerland; ^4^Institute of Biology and Ecology, Pavol Jozef Šafárik UniversityKošice, Slovakia

**Keywords:** GGPPS, isoprenoids, paralogs, specialized metabolism, subfunctionalization

## Abstract

Multiple geranylgeranyl diphosphate synthases (GGPPS) for biosynthesis of geranylgeranyl diphosphate (GGPP) exist in plants. GGPP is produced in the isoprenoid pathway and is a central precursor for various primary and specialized plant metabolites. Therefore, its biosynthesis is an essential regulatory point in the isoprenoid pathway. We selected 119 GGPPSs from 48 species representing all major plant lineages, based on stringent homology criteria. After the diversification of land plants, the number of *GGPPS* paralogs per species increases. Already in the moss *Physcomitrella patens*, GGPPS appears to be encoded by multiple paralogous genes. In gymnosperms, neofunctionalization of GGPPS may have enabled optimized biosynthesis of primary and specialized metabolites. Notably, lineage-specific expansion of GGPPS occurred in land plants. As a representative species we focused here on *Arabidopsis thaliana*, which retained the highest number of GGPPS paralogs (twelve) among the 48 species we considered in this study. Our results show that the *A. thaliana GGPPS* gene family is an example of evolution involving neo- and subfunctionalization as well as pseudogenization. We propose subfunctionalization as one of the main mechanisms allowing the maintenance of multiple *GGPPS* paralogs in *A. thaliana* genome. Accordingly, the changes in the expression patterns of the *GGPPS* paralogs occurring after gene duplication led to developmental and/or condition specific functional evolution.

## Introduction

Isoprenoids represent the largest group of biologically active specialized metabolites in plants. Many have roles in protecting the plants against pathogens and herbivores or conversely they attract pollinators and seed-dispersing animals. (Bouvier et al., 2005). Other isoprenoids have important roles in photosynthesis and respiration or as hormones (abscisic acid, brassinosteroids, cytokinins, gibberellic acid, strigolactones) in development and growth regulation (Bouvier et al., 2005; Liang, 2009; Vranová et al., 2012).

In spite of their broad diversity of functions and structures, the biosynthesis of all isoprenoids in plants invariably requires two five-carbon (C5) building units: the isopentenyl diphosphate (IPP) and its isomer dimethylallyl diphosphate (DMAPP) (Liang et al., 2002; Hsieh et al., 2011; Vranová et al., 2013). In plants, the mevalonic acid pathway (MVA) produces cytosolic IPP, and the methylerythritol pathway (MEP) produces IPP and DMAPP in plastids (Goldstein and Brown, 1990; Rohmer, 1999; Rodríguez-Concepción and Boronat, 2002). The MVA and MEP pathways are linear step enzymatic reactions until the synthesis of the allylic prenyl diphosphates. Then, prenyl diphosphate synthases catalyze chain elongation reactions by coupling IPP to DMAPP producing allylic prenyl diphosphates of different length (Vranová et al., 2013). Most of the essential plant isoprenoids are derived from the C15 and C20 allylic prenyl diphosphates farnesyl-PP (FPP) and geranylgeranyl-PP (GGPP), whose pools represent nodes of the major metabolic branch points in the isoprenoid synthesis (Vranová et al., 2011).

In plants, the enzymes catalyzing the steps upstream of GGPP biosynthesis are encoded either by single copy genes or by pairs of genes (Goldstein and Brown, 1990; Rodríguez-Concepción and Boronat, 2002; Closa et al., 2010; Vranová et al., 2013). Intriguingly, at the GGPP branch point, a high number of genes encoding GGPP synthase is predicted for plant genomes, reaching up to 12 members per species (PLAZA, http://bioinformatics.psb.ugent.be/plaza/).

Multiple gene copies result from duplication events, which can involve individual genes, chromosomal segments, or entire genomes (whole-genome duplication, WGD). Such genes descend from a common ancestor and are homologous (Innan and Kondrashov, 2010). Homologous genes are further classified into paralogs, which are related by duplication events and orthologs, which are genes in different species that evolved from a common ancestor through speciation events (Fitch, 1970). Whereas orthologs tend to share similar functions, paralogs tend to have different roles (Studer and Robinson-Rechavi, 2009). Following duplication, one of the outcomes for a paralog is to accumulate inactivating mutation and become a pseudogene (Innan and Kondrashov, 2010). Alternatively, paralogs are preserved in the genome, particularly if they confer selective advantages. For example, one gene may retain the ancestral function whereas the other undergoes accelerated evolution to acquire a new function (“neofunctionalization”) (Innan and Kondrashov, 2010). Or both paralogous copies might specialize and retain only distinct subsets of the ancestral gene function (“subfunctionalization”), which may increase the fitness of the organism (Lynch and Conery, 2000; Lynch and Force, 2000).

Although biosynthesis of GGPP is an essential step in the isoprenoid pathway providing the common precursor for key metabolic pathways involved in both primary and specialized metabolism, to date, our understanding of specific function of individual geranylgeranyl diphosphate synthases (GGPPS) paralogs is limited (Ament et al., 2006; Jassbi et al., 2008; Schmidt et al., 2010). Reports on basic characterization of individual GGPPS isozymes from *A. thaliana* date back more than a decade ago (Zhu et al., 1997a,b; Okada et al., 2000), being completed only in the recent years (Wang and Dixon, 2009; Beck et al., 2013). This emphasizes the difficulties of studying multiple paralog gene families *in vivo*.

According to our current knowledge, 10 GGPPS (GGPPS1-GGPPS4 and GGPPS6-GGPPS11) out of 12 predicted paralogs from *A. thaliana* are functional, i.e., GGPP is the major product they synthesize *in vitro* and/or they complement *E. coli* strains engineered to synthesize lycopene but lacking GGPPS activity (Zhu et al., 1997a,b; Okada et al., 2000; Wang and Dixon, 2009; Beck et al., 2013).

Furthermore, the GGPPSs from *A. thaliana* reside in distinct subcellular compartments and have distinct expression patterns during plant development. GGPPS1 is targeted to mitochondria, GGPPS3 and GGPPS4 to the ER, GGPPS2 and GGPPS6-GGPPS11 to plastids (Zhu et al., 1997a,b; Okada et al., 2000; Wang and Dixon, 2009; Beck et al., 2013). *GGPPS11* is ubiquitously and abundantly expressed, mainly in photosynthetically active tissues (Okada et al., 2000; Beck et al., 2013), likely providing the GGPP substrate for biosynthesis of essential photosynthesis-related isoprenoid compounds such as chlorophylls, carotenoids, phylloquinones or plastoquinones. *GGPPS1*-*GGPPS10* expression is different during plant development. These paralogs are expressed predominantly in specific root or seed tissues (Beck et al., 2013). Additionally, *GGPPS5* was proposed to be a pseudogene based on sequence analysis (Beck et al., 2013), whereas GGPPS12, the most distant paralog from all predicted GGPP synthases in *A. thaliana*, does not have GGPP synthase activity (Okada et al., 2000; Wang and Dixon, 2009; Beck et al., 2013). However, GGPPS12 seems to be active as a heterodimer and together with GGPPS11 can synthesize geranyl diphosphate (GPP) (Wang and Dixon, 2009).

The localization in different subcellular compartments as well as the distinct expression pattern suggest specific roles for the GGPPS paralogs during *A. thaliana* development. Yet, the biological significance of a highly expanded GGPP branch point and the relationship between the sequence and function of the GGPPS isozymes is not fully understood.

Here, we investigate the evolutionary relationships and molecular characteristics of the GGPPS homologs in plants using a combination of computational analyses and integration with meta-analysis of existing data sets. We identified the GGPPS homologs from 48 plant species representing major plant lineages (green algae, mosses, gymnosperms, and angiosperms) and inferred their evolutionary relationships. We show that multiple within-species GGPPS paralogs exist in several land plants lineages, particularly in angiosperms. The presence of GGPPS paralogs in the moss *P. patens* suggests that GGPPS duplicated early after the diversification from green algae. In gymnosperms, molecular changes in the GGPPS protein domain may have enabled the transition from biosynthesis of primary GGPP-derived compounds to specialized GPP (geranyl diphosphate) metabolites, which play roles in plant-environment interactions. In land plants, a lineage-specific expansion trend of GGPPS is observed.

We have particularly focused on the model plant *A. thaliana* whose nuclear genome retained 12 GGPPS (Lange and Ghassemian, 2003), the highest number of GGPPS paralogs in plants whose genomes have been sequenced to date. Our results suggest that the expansion of the GGPPS family in *A. thaliana* occurred at distinct time points in evolution and by different duplication mechanisms. *GGPPS12, GGPPS2-4*, and *GGPPS11* diverged first. *GGPPS2-4* and *GGPPS11* arose during the most recent WGD event that occurred in *A. thaliana*. In contrast, the most recently diverged paralogs (*GGPPS6, GGPPS7, GGPPS9*, and *GGPPS10*) arose by tandem and segmental genome duplication. Moreover, we hypothesized that if the GGPPS paralogs from *A. thaliana* are not redundant, their persistence in the genome might be attributed to acquired neo- or subfunctionalization. To test this hypothesis, we have inferred the expression states of individual *GGPPS* during plant development. Subsequently, we have mapped these expression states onto the phylogenetic tree of the GGPPS paralogs from *A. thaliana* and inferred the most parsimonious expression pattern of the ancestral GGPPS gene. A statistically significant correlation of sequence and expression divergence substantiated our hypothesis of subfunctionalization in terms of differential expression pattern.

## Materials and methods

### Sequence retrieval and phylogenetic analysis

To study the phylogeny of the GGPPS family a rooted maximum-likelihood (ML) tree from 119 homologous protein sequences spanning 48 plant genomes was reconstructed as follows. First, the homologs were selected by searching sequences (i.e., protein sequences including targeting peptides) similar to the 12 predicted GGPPS proteins from *A. thaliana* in the UniProtKB database (The UniProt Consortium, 2009) augmented with the *A. lyrata* genome retrieved from Ensembl Plants v3 (Kersey et al., 2010). The current protein model for GGPPS5 reposited at TAIR v.10 (http://www.arabidopsis.org/tools/bulk/sequences/index.jsp), which proposes that the translation could be initiated at an alternative start codon, resulting in a protein that lacks a plastidial targeting sequence at the N terminus but has a conserved polyprenyl synthase domain was used (Beck et al., 2013).

To qualify as a homolog, sequences had to exceed a Dayhoff alignment score of 130 to all GGPPS from *A. thaliana* proteins using Darwin's Align function (Gonnet et al., 2000). From this set of homologs, a multiple sequence alignment (MSA) was reconstructed (Supplementary Dataset 1) using the Mafft FFT-NS-2 method (Katoh and Toh, 2008). From the resulting MSA, a maximum likelihood tree was reconstructed using the PhyML 3.0 software (Guindon and Gascuel, 2003; Guindon et al., 2009). The default parameters were kept, i.e., we have used the *LG* amino acid substitution matrices (Le and Gascuel, 2008), without invariant sites and with four discrete rate categories chosen according to an estimated gamma shape parameter. The reconstruction was done 50 times from different starting topologies and the overall highest scoring reconstruction was kept for the subsequent analysis. Branch support values were computed using the approximate likelihood ratio test (aLRT) (Anisimova and Gascuel, 2006). To root the phylogenetic tree, a parsimony-based method was used (Berglund-Sonnhammer et al., 2006). In brief, from all possible rootings the tree which minimized the number of implied duplication events and gene losses was chosen. Finally, to infer internal nodes of the tree as speciation or duplication nodes we used the species overlap method, which does not assume a particular species phylogeny (Van Der Heijden et al., 2007). In brief, at every inner node of the gene tree, the overlap of species that are present in each of the two subtrees were counted. In cases one species appeared on both sides of the gene tree, a duplication was inferred; else a speciation event was inferred.

Relative divergence dates of the *GGPPS* paralogs from the Arabidopsis lineage were estimated using Bayesian phylogeny reconstruction with the BEAST 1.6.1 and the BEAGLE software (Drummond et al., 2006). From the previously computed MSA, taxa outside the relevant Arabidopsis lineage were removed and the syntenic orthologs from *Carica papaya* were included (CP00020G01300 and CP00158G00190; PGDD database, http://chibba.agtec.uga.edu/duplication/). The aligned amino acid sequences were mapped to their corresponding codon sequences. Using the *ECM* + *F* + ω + 2_K_ codon substitution model (Kosiol et al., 2007) in the BEAST software, proposition trees for the tree sampling process were generated by a Yule speciation process using an uncorrelated relaxed clock model with logNormal distribution (Drummond et al., 2006). To calibrate the evolutionary timescale, the following normal distribution priors from the literature on the age of two evolutionary events were used: the *A. thaliana* and *A. lyrata* split was set to 13 ± 3 mya (Beilstein et al., 2010) and the stem lineage subtending the eudicot crown group was set to 130 ± 5.5 mya (Davies et al., 2004). The Markov Chain Monte Carlo (MCMC) chain-length was set to 8 × 10^6^. The first 1% of the trees was discarded as burn-in. The TreeAnnotator module from the BEAST software was used to create the consensus trees.

### Expression analysis

The expression profile map of the GGPPS paralogs from *A. thaliana* was assembled based on ATH1 22K Affymetrix GeneChip microarray data generated by the AtGenExpress Consortium (http://www.weigelworld.org/resources/microarray/AtGenExpress). The AtGenExpress normalized datasets “tissue extended plus” was retrieved from the Bio-Array Resource website (BAR, www.bar.utoronto.ca). Only experiments using wild-type plants were considered. The probesets for the majority of the GGPPS paralogs are specific to their corresponding transcript, except for *GGPPS6* and *GGPPS7* whose transcripts are ambiguously recognized by the same probeset (258121_s_at) due to their high nucleotide sequence similarity. The common expression profiles for these two genes will be referred in figures with the notation “*GGPPS6/7.*” Expression values below a threshold of 2.5 (log2 scale) were considered as not detectable on the microarray (Schmid et al., 2005; Beck et al., 2013). Hierarchical agglomerative clustering with a threshold set at a tree height *h* = 0.35 (equivalent to a Pearson correlation coefficient of 0.65) was used to estimate the number of clusters and their composition. The cluster analysis was conducted in R (R Development Core Team, 2010).

### Ancestral state reconstruction and statistical analysis

The ancestral state reconstruction and random permutations were performed with the Mesquite system for phylogenetic computing version 2.75 (Maddison and Maddison, 2011). The character matrix was generated by discretizing the expression clusters, i.e., each expression cluster is assigned to a distinct character state. The ancestral state reconstruction was performed under a parsimony model assuming an unordered model in which all state changes are weighed equally. To evaluate the statistical significance of an observed parsimony score, the data were randomly permuted by reshuffling the discrete states among taxa 1 × 10^4^ times and calculating the parsimony score for each repetition. The *p*-value was estimated from the distribution of the random parsimony scores, as the fraction of random scores (including the observed score) less than or equal to the observed score: *p* = (1+*k*)/*n* where *k* is the number of replications with less or as many steps than the actual observed data and *n* is the total number of replications. A significant phylogenetic signal was observed at a *p-*value smaller than 0.05 (Faith and Cranston, 1991; Wahlberg, 2001).

## Results and discussion

### The number of *GGPPS* gene paralogs increases during the evolution of plant functional complexity

We have investigated the phylogenetic relationships among GGPPSs from plants to infer evolutionary mechanisms leading to the formation and maintenance of multiple gene copies particularly within the *A. thaliana* genome, which had retained the highest number of paralogs (twelve).

In total, 119 homologous protein sequences exceeding a Dayhoff alignment score of 130 to all GGPPS from *A. thaliana* (see Materials and Methods) were identified and selected for the phylogenetic tree reconstruction. The selected GGPPS homologs represent 48 plant genomes ranging from green algae and mosses to gymnosperms and angiosperms (Supplementary Table 1).

**Figure 1:**
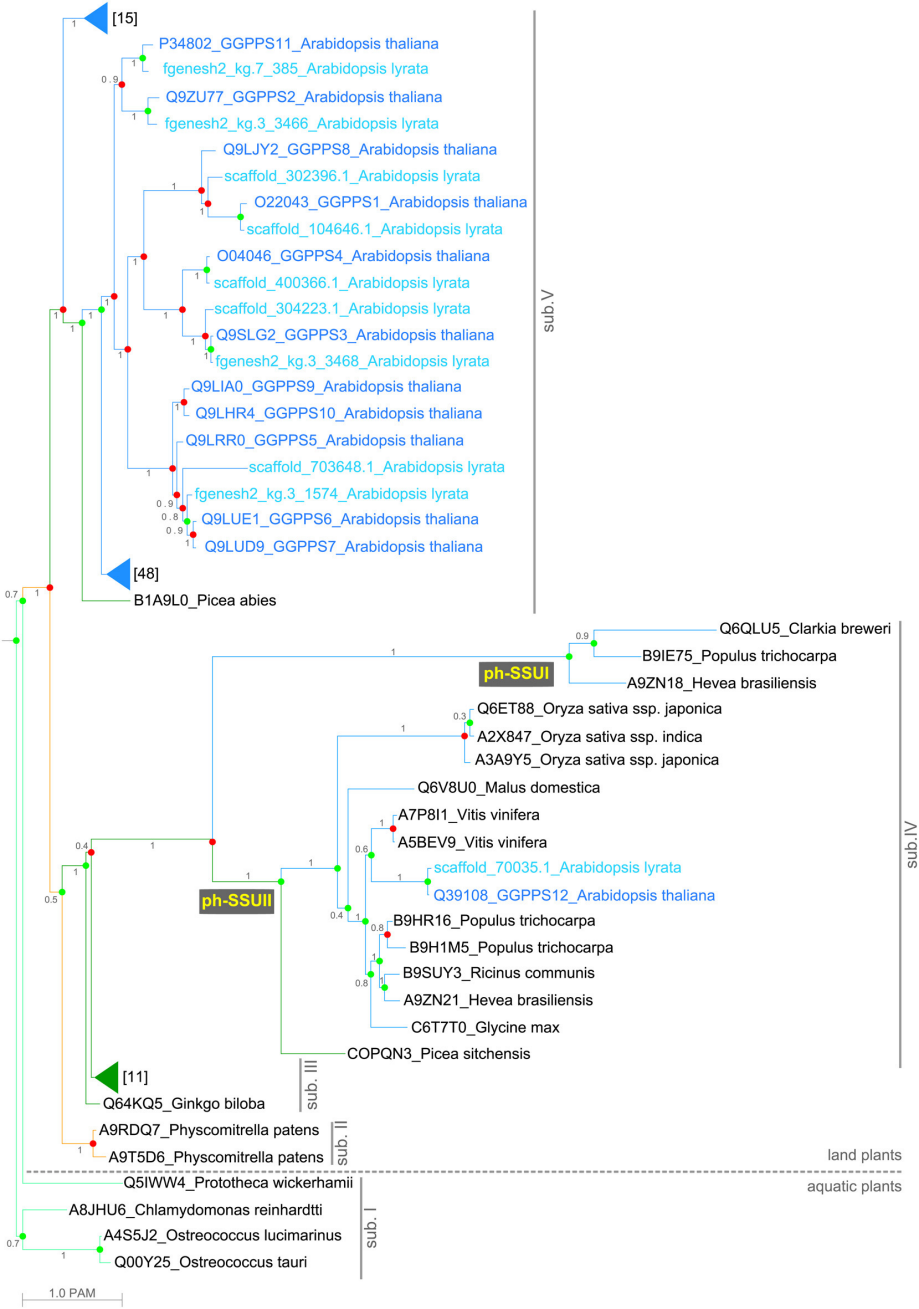
**Maximum likelihood consensus tree of the 119 GGPPS homologs from plants**. Posterior probabilities are shown next to the branches. Branch lengths correspond to evolution distances (see Materials and Methods). Duplication (red dots) and speciation (green dots) events are shown at nodes. The tree is divided into five classes (sub. I–V). Branch colors represent the major plant lineages: spring green, green algae; orange, mosses; dark green, gymnosperms; and blue- angiosperms. Branches holding homologs from gymnosperms and angiosperms are collapsed and the number of homologs in each collapsed group is shown. The homologs from the Arabidopsis lineage are shown: in blue-*A. thaliana*, in cyan-*A. lyrata*.

The GGPPS phylogenetic tree revealed five main subfamilies, referred here to as sub. I to sub.V (Figure 1). Plant-specific *GGPPS* genes might have originated from an ancestral copy that was present in the common ancestor of land plants and green algae. This is in agreement with earlier publications proposing that all trans-isoprenyl diphosphate synthases, an enzyme class including the GGPPSs, are derived from a common ancestral gene whose precise identity as archaeal or bacterial homolog is not fully elucidated to date (Chen et al., 1994; Tachibana et al., 2000). Early after the diversification of land plants, the number of *GGPPS* paralogs per species increases and already in the moss *P. patens* GGPPS appears to be encoded by multiple gene paralogs. Furthermore, the phylogenetic analysis showed lineage-specific expansion and divergence events occurring in land plants (Figure 1 and Supplementary Figure 1). The increase in the predicted number of GGPPSs per species mirrors the increase in complexity of the species. From one GGPPS in green algae (sub. I), three in mosses (sub. II and sub. V) and one to four in gymnosperms (sub. III–V), the number of GGPPS paralogs per species reaches a maximum of twelve copies within angiosperms in *A. thaliana* (sub. V; Supplementary Table 1).

### The molecular evolution of the polyprenyl synthase domain enables the neofunctionalization of GGPPS

To gain further insights in molecular changes underlying the evolution of the GGPPS homologs in plants, we have analyzed the evolution of the characteristic polyprenyl synthase domain (Liang et al., 2002). The GGPPS polyprenyl synthase domain has a first aspartate rich motif, FARM (DDxxxxD; x is any amino acid) and a second aspartate rich motif, SARM (DDxxD; x is any amino acid), which are involved in IPP and DMAPP substrate binding and are critical for GGPP biosynthesis (Liang et al., 2002).

Whereas GGPPSs are typically active as homodimers (Vandermoten et al., 2009), heterodimeric complexes between functional GGPPS and SSUI and SSUII (heterodimeric GPP synthase small subunit I and II, respectively) synthesizing GPP have been reported (Burke et al., 1999; Tholl et al., 2004; Wang and Dixon, 2009). SSUI lost both aspartate rich motifs but has two conserved CxxxC motifs (where x is any hydrophobic amino acid) (Tholl et al., 2004). SSUII has conserved FARM and two CxxxC motifs (Burke et al., 1999; Wang and Dixon, 2009). In heterodimeric complexes between functional GGPPS and SSUII, the CxxxC motifs were shown to be important for physical interaction between subunits. Furthermore, such complexes were shown to be able to produce, with increased efficiency, GPP (Wang and Dixon, 2009). GPP can be also produced by homodimeric GPS (geranyl diphosphate synthase) (Hsiao et al., 2008; Schmidt and Gershenzon, 2008). Interestingly, a protein from *A. thaliana* initially classified as GPS (At2g34630; (Bouvier et al., 2000; Van Schie et al., 2007)), which lost the CxxxC motifs but has conserved FARM and SARM, was shown to produce medium (C25) to long (C45) chain isoprenoid products, and was therefore renamed as polyprenyl pyrophosphate synthase (AtPPPS; Hsieh et al., 2011).

**Figure 2:**
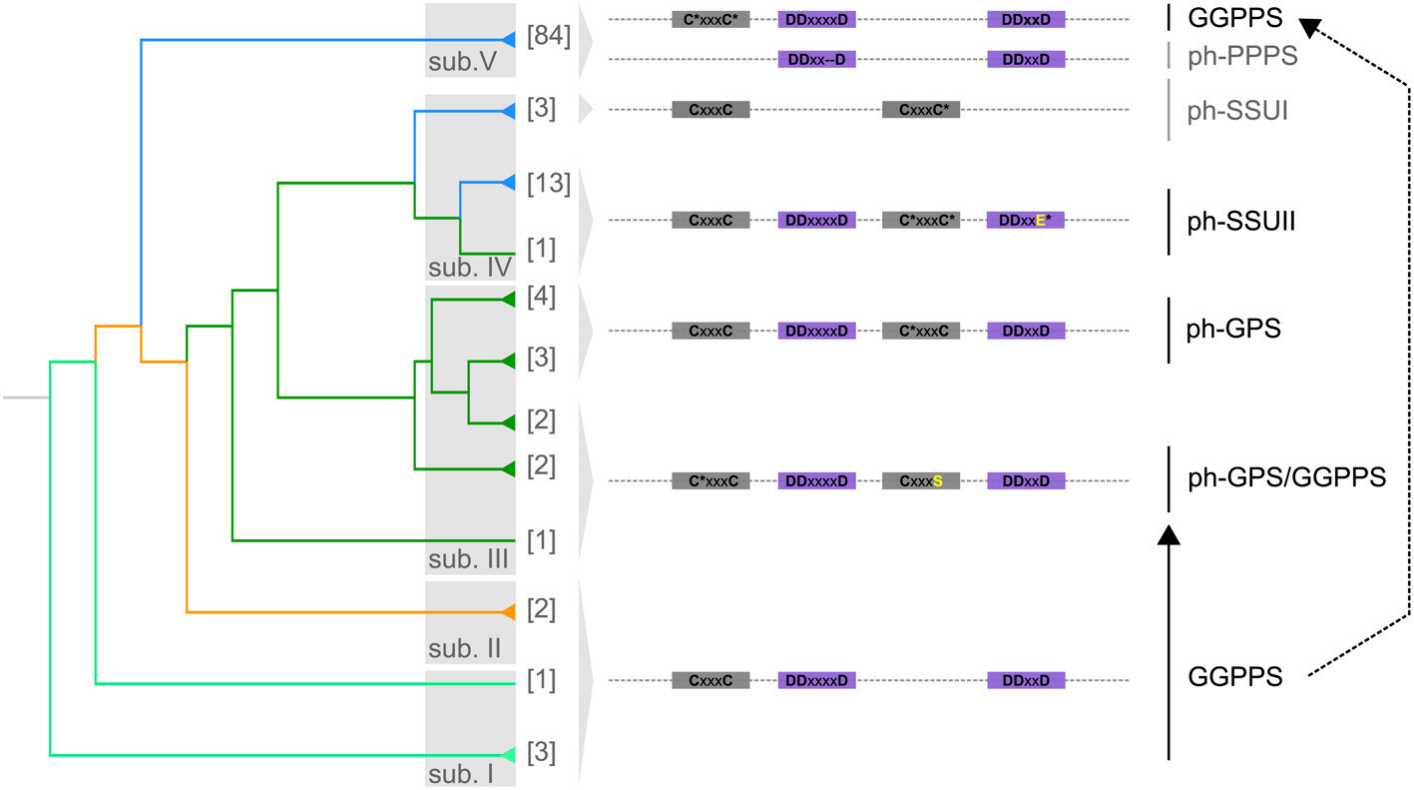
**Molecular evolution of the polyprenyl synthase domain**. The summarized phylogenetic tree of GGPPS from plants is shown. Branches holding more than one homolog are collapsed and the number of homologs is shown. The five classes (sub. I–V) of GGPPS homologs in plants are shown. Branch colors represent the major plant lineages: spring green, green algae; orange, mosses; dark green, gymnosperms; and blue, angiosperms. The representative polyprenyl synthase motifs for each of the five classes are shown: the two CxxxC motifs in gray and FARM, SARM in purple. Asterisk (^*^) indicates variable amino acid residues (Supplementary Figure 1 and Supplementary Table 2). ph-GPS: putative homologs of GPS, ph-GPS/GGPPS: putative homologs of the bifunctional GPS/GGPPS. A prototype of the second CxxxC motif (CxxxS; the serine residue is shown in yellow) appears to have been acquired in a common ancestor of gymnosperms. ph-PPPS: putative homologs of polyprenyl pyrophosphate synthase. ph-SSUI and ph-SSUII: putative homologs of the small subunit (SSU) of heterodimeric GPS. Ph-SSUI proteins have lost the two conserved FARM and SARM motifs. None of the ph-SSUII proteins have a conserved SARM (the variable mutated amino acid residue is shown in yellow) indicating loss of GGPPS capacity.

The GGPPS homologs from sub. I, II and V have highly conserved FARM, SARM and one CxxxC motif (Figure 2 and Supplementary Figure 2). Homologs from *A. thaliana* with such protein domain structure were shown to be active as homodimers and produce GGPP (Okada et al., 2000; Wang and Dixon, 2009; Beck et al., 2013).

Several homologs from sub. V, have lost the CxxxC motif (Figure 2). Such proteins, referred here to as ph-PPPS (putative homologs of polyprenyl pyrophosphate synthase) retain solely FARM and SARM motifs and are found at *d* = 7.03 distance from root supporting their rapid divergence (Supplementary Figure 1 and Supplementary Table 2). The polyprenyl pyrophosphate synthase (AtPPPS, At2g34630) from *A. thaliana*, which can synthesize medium (C25) to long (C45) chain isoprenoid products, has a similar domain structure as the ph-PPPS proteins (Hsieh et al., 2011).

Within sub. III that is found exclusively in gymnosperms, in addition to the conserved FARM and SARM, a prototype of a second CxxxC motif (CxxxS) appears to have been acquired in a common ancestor of Ginkgo, Taxus, Abies and Picea species (Figure 2, Supplementary Figure 1 and Supplementary Table 2). A protein with similar domain structure was recently reported to be bifunctional, producing both GPP and GGPP (Schmidt et al., 2010). GPP is the precursor for biosynthesis of monoterpenoids, a class of specialized metabolites which play roles in pollination, seed dispersal and defense mechanisms (Bohlmann and Croteau, 1999). This suggests that the molecular changes in the protein domains of orthologs found in this class may have enabled the transition from biosynthesis of primary GGPP-derived compounds to specialized GPP-derived metabolites. In Abies and Picea species, mutation of the serine residue to cysteine resulted in a conserved second CxxxC motif (Figure 2, Supplementary Figure 1 and Supplementary Table 2). The homolog B1A9K6 from *Picea abies* (Supplementary Table 2), which retains two conserved CxxxC concomitant with FARM and SARM, was shown to produce only GPP (Schmidt and Gershenzon, 2008).

The GGPPS homologs from sub. IV appear to have experienced faster sequence divergence compared to sub. III, indicated by the branch length (Figure 1). Both FARM and SARM are either missing or SARM is mutated in sub. IV but both CxxxC motifs are present (Figure 2). Sub. IV comprises of GGPPS from monocots and dicots and one homolog from gymnosperms, most of them being uncharacterized to date (Figure 1). Sub. IV is further comprised of two subclasses referred to here as ph-SSUI and ph-SSUII, i.e., putative homologs of the small subunit (SSU) of heterodimeric GPS (Tholl et al., 2004; Wang and Dixon, 2009). Members of both ph-SSUI and ph-SSUII were shown to be active not as GGPPS but as SSU in heterodimeric GPS complexes, producing the GPP (Tholl et al., 2004; Wang and Dixon, 2009). Interestingly, ph-SSUI members are mainly found in flowering plant species (Figure 2 and Supplementary Table 2). They have lost both aspartate rich motifs (Figure 2), likely rendering them inactive as homodimeric enzymes. Consistently, the Q6QLU5 homolog from *Clarkia breweri* (Figure 1; ph-SSUI) does not produce GGPP (Tholl et al., 2004). A homolog from *Antirrhinum majus*, with similar protein domain structure was shown to form heterodimeric GPS complexes with functional GGPPS and synthesize GPP as main product in reproductive organs (Tholl et al., 2004). In summary, this subclass of proteins with the unique motif organization (lacking both SARM and FARM but retaining both CxxxC motifs) seems to be responsible for monoterpenoids precursor biosynthesis in reproductive plant organs. Members of the ph-SSUII branch from sub. IV have intact FARM but mutated SARM (Figures 1, 2 and Supplementary Table 2). The GGPPS12 homolog from *A. thaliana* has such a protein domain structure and consequently, is unable to produce GGPP (Okada et al., 2000). Furthermore, similarly to characterized proteins from ph-SSUI (Wang and Dixon, 2009), GGPPS12 forms heterodimeric complexes with GGPPS11 and redirects biosynthesis toward GPP (Okada et al., 2000; Wang and Dixon, 2009). In contrast to ph-SSUI homologs, which are likely to play a role in monoterpenoid biosynthesis mainly in reproductive organs, members of the ph-SSUII were proposed, based on their expression pattern, to constitutively participate in GPP biosynthesis during plant development (Wang and Dixon, 2009).

Taken together, GGPPS homologs with canonical protein domain structure are present in all major plant lineages investigated here. Early after the diversification of land plants, duplication events led to multiple GGPPS genes per species, providing raw material for evolutionary change. Yet, with the divergence of land plants their functional complexity and need for defense strategies also diversified.

By neofunctionalization of GGPPS, novel heterodimeric GPS complex formation capacity, and thereby the GPP biosynthesis was enabled by the acquisition of a second CxxxC motif that likely occurred in the ancestor of gymnosperms. GPP serves as precursor of monoterpenes, which are involved in direct defense mechanisms against herbivores or pathogens, they can indirectly protect plants by attracting predators of attacking herbivores, or they can be emitted from floral tissues to attract pollinators (Pichersky and Gershenzon, 2002; Chen et al., 2003; Keeling and Bohlmann, 2006). Members of the ph-PPPS (sub. V), whose protein domains are similar to the AtPPPS from *A. thaliana* (Bouvier et al., 2000; Hsieh et al., 2011) are likely another example of neofunctionalization. They have lost the two CxxxC motifs and in *A. thaliana*, this enzyme is able to generate multiple products with medium to long chain lengths (C25–C45) (Hsieh et al., 2011).

### Lineage-specific expansion of *GGPPS* is most evident in arabidopsis

Duplication events leading to lineage-specific expansion of GGPPS (i.e., no discernible ortholog in closely related species) occurred in land plants (Supplementary Figure 1). The most prominent example of lineage-specific expansion, with respect to our taxon sampling, is found in the Arabidopsis lineage where, the high GGPPSs sequence similarity determines their clustering in the phylogenetic tree (Figure 1). The majority of the GGPPS paralogs in *A. thaliana* and its closest relative *A. lyrata* are found in the same clade and are more similar to each other than to homologs from other species, which is supported by the high branch support values (aLRT ≥ 0.8). In particular, *A. thaliana* encodes the largest number of paralogs from the species investigated here, including a unique set of GGPPSs (GGPPS6, GGPPS7, GGPPS9, and GGPPS10) found only in this species (Figure 1).

Lineage-specific expansion followed by subfunctionalization is known to be an important mechanism for diversification of gene function (Lespinet et al., 2002; Nowick and Stubbs, 2010). For example, the expression of lineage-specific genes in *A. thaliana* was observed to be confined to fewer tissues, where they are involved particularly in abiotic stress responses (Donoghue et al., 2011).

The expression of the *GGPPS* paralogs specific to *A. thaliana* is under strict developmental control, being expressed in specific tissues and at distinct time during plant development (Beck et al., 2013). For example, *GGPPS6* is expressed only in the meristematic zone of the root tip (columella and lateral root cap), whereas *GGPPS10* expression is distributed over the length of the root but not in the root tip (Beck et al., 2013). Together, these indicate that LSG GGPPS paralogs may have special function only at particular stages during plant development and possibly in response to external environmental signals.

### Subfunctionalization maintains multiple *GGPPS* paralogs in the *a. thaliana* genome

Multiple *GGPPS* paralogs might have been maintained in the genome of *A. thaliana* due to the divergence in their expression patterns. There should be no selective constraints blocking this divergence as long as the initial expression pattern of the ancestral gene is maintained. Thus, we expect that the GGPPS paralogs may have specialized functions in *A. thaliana* according to their expression profiles.

**Figure 3:**
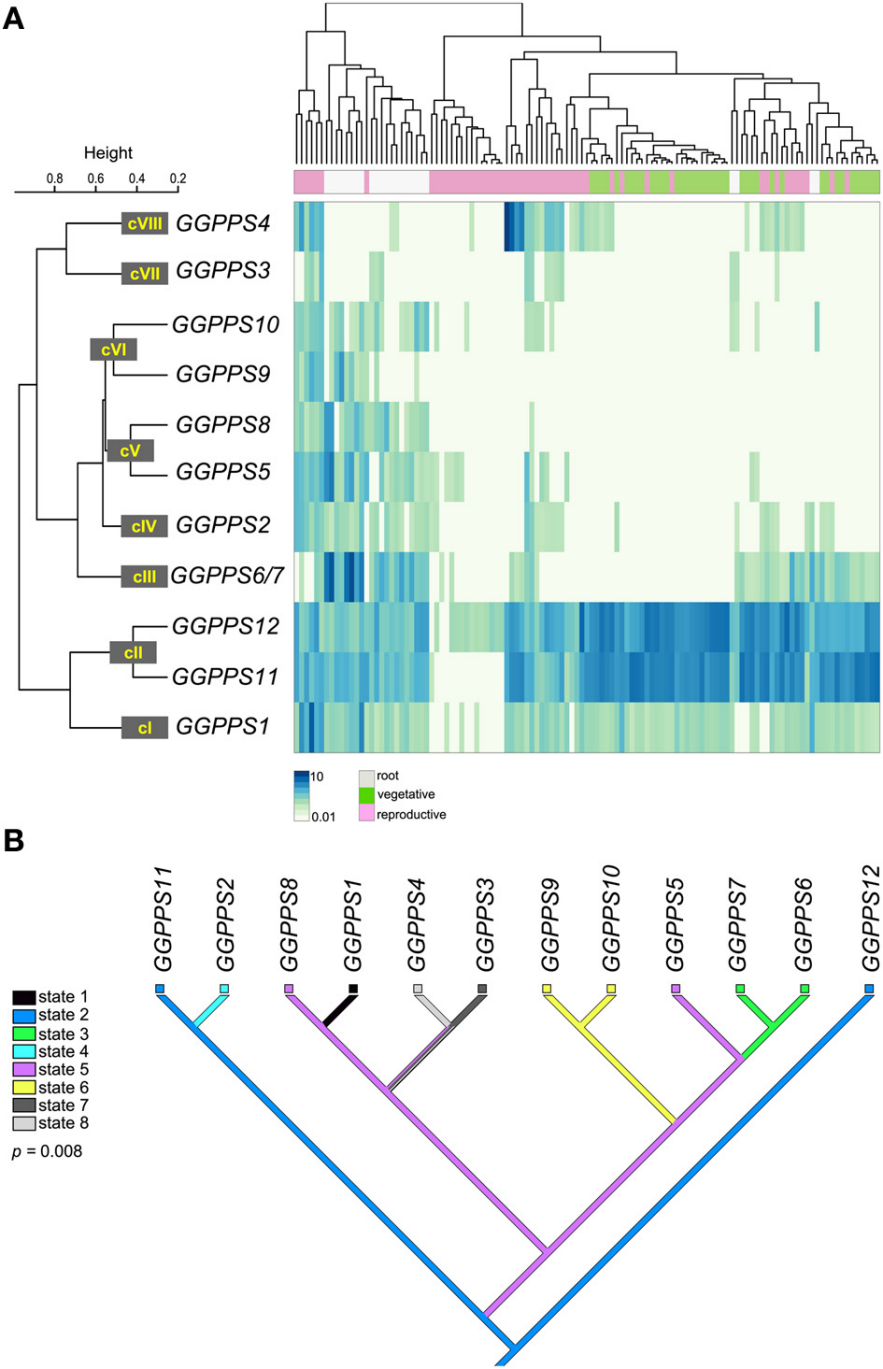
**Expression pattern analysis of the *GGPPS* genes from *A. thaliana* and ancestral states reconstruction. (A)** The clustering of microarray expression data is shown as heatmap. The expression clusters (cI-VIII) of the *GGPPS* paralogs identified based on Pearson correlation coefficients with a threshold set to *r* = 0.65 (see Materials and Methods) are shown. The various organ and tissue samples were assigned to three major classes: root (white box), vegetative (green box; includes samples from the seedlings, rosette leaves, stems, and cauline leaves) and reproductive (pink box; includes samples from flowers and seeds). **(B)** The phylogenetic reconstruction of ancestral expression states using parsimony is shown. The colors corresponding to each expression state (state 1–8) are shown in the legend. Colored boxes are shown at terminal branches indicating the observed expression pattern cluster. Branches with multiple colors are associated with several possible expression states.

To test this hypothesis we mapped *A. thaliana GGPPSs* expression data onto the phylogenetic tree and reconstructed the ancestral expression states (Figure 3). Using a comprehensive dataset for gene expression during *A. thaliana* development (see Materials and Methods) we defined eight expression clusters containing the *GGPPS* paralogs referred to as cI-VIII (Figure 3A). Next, we mapped the expression clusters as discrete states onto the phylogenetic tree of the *GGPPS* paralogs in *A. thaliana.* The reconstruction of ancestral expression states was performed using the Mesquite v2.75 system for phylogenetic computing (Maddison and Maddison, 2011), which allows the inference of the most likely hypothetical expression states for the ancestral gene under a maximum parsimony model (Figure 3B). The expression states (state 1–8) are shown as colored boxes at the terminal branches. A change in color between sister branches indicates a putative divergence in the expression pattern of the paralog.

The ancestral expression pattern, state 2, is represented by an ubiquitous gene expression during plant development (Figure 3B). From an evolutionary perspective, ubiquitous expression is characteristic to housekeeping genes, which are generally associated with slower evolutionary rates (Hurst and Smith, 1999; Koonin, 2009). Thus, housekeeping genes are less likely to experience divergence of their expression pattern. As expected, the parsimony reconstruction supports a ubiquitous expression pattern (state 2) of the ancestral *GGPPS* in *A. thaliana* during plant development. *GGPPS11* and *GGPPS12* represent expression state 2, while the expression pattern of the remaining *GGPPS* paralogs appears to be under developmental control. As such, the expression pattern of the *GGPPS* gene family during development diverged during several rounds of duplication. Some of the emerging expression states are clade specific (state 6; Figure 3B). However, there is also an example of same or similar expression pattern that appears to have emerged at different positions in the tree. For example, *GGPPS5* and *GGPPS8* are part of the same class V as they have a similar expression pattern (*r* = 0.76) but are found in distinct phylogenetic clades (Figure 3). This suggests that these two paralogs may have independently acquired or lost similar cis-regulatory elements responsible for the regulation of expression during development. Furthermore, several paralogs share a similar expression pattern, which likely reflects the short time since their divergence as in the case of *GGPPS9* and *GGPPS10* (Figure 3B).

To exclude random events, we evaluated the statistical significance of the correlation between sequence and expression divergence by performing a permutation test in which the expression states were randomly reshuffled. Subsequently, we performed 10,000 ancestral states reconstructions and compared the observed parsimony score against the random distribution from which we calculated the *p*-values. The number of steps required in the random distribution ranged from 7 to 10 in the case of the ancestral states reconstruction of the expression patterns during development. The observed parsimony score of 7 steps indicates non-random distribution that is supported statistically by a permutation *p*-value of 0.008. Therefore, during the evolution of the *GGPPS* gene family in *A. thaliana* the divergence in expression pattern appears to be coupled, at least partially, to sequence divergence.

*GGPPS12* and *GGPPS11* genes have an ancestral, ubiquitous expression pattern (Figure 3) that may reflect their requirement as housekeeping genes encoding for GGPPS and SSUII, respectively. *GGPPS5* was proposed to encode a pseudogene based on the sequence analysis, which identified a frame shift mutation rendering translation of a truncated GGPPS protein (Beck et al., 2013). Nevertheless, probe based hybridization arrays were able to detect specific expression of *GGPPS5* gene in different organs of *A. thaliana* (Figure 3) indicating that *GGPPS5* is an expressed pseudogene also known as ghost pseudogene (Zheng and Gerstein, 2007). As a ghost pseudogene, *GGPPS5* could play a role in regulating the function of closely related paralogs, for example by competing for the cellular RNA degradation machinery (Hirotsune et al., 2003).

*GGPPS1* and *GGPPS2* are expressed ubiquitously in all plant organs, but at much lower levels than *GGPPS11* and *GGPPS12* (Figure 3A; Beck et al., 2013). *GGPPS3, GGPPS4*, and *GGPPS8* have a mosaic of expression patterns during the plant development. *GGPPS3* and *GGPPS4* are predominantly expressed in reproductive organs and root vasculature, whereas *GGPPS8* is specifically expressed in the outer cell layers above the mitotically active area of the root (Figure 3A; Beck et al., 2013). The expression of the *GGPPS* paralogs specific to *A. thaliana* (*GGPPS6, GGPPS7, GGPPS9*, and *GGPPS10*) is confined to particular tissues (Figure 3A; Beck et al., 2013), suggesting that they might play a role only at defined developmental stages and/or in fine tuning adaptation to specific conditions.

Collectively, in addition to neofunctionalization of GGPPS, another mechanism allowing the maintenance of multiple duplicated *GGPPS* paralogs in the *A. thaliana* genome appears to be their subfunctionalization in terms of differential expression pattern during plant development.

### The duplication timing reveals a correlation between age and expression pattern of the *GGPPSs* from *a. thaliana*

*A. thaliana* is an ancient polyploid that through evolutionary history experienced three major whole genome duplication events termed γ, β, and α in the order of their occurrence (Bowers et al., 2003). Species such as *Carica papaya* that have not experienced any other whole genome duplication since the γ-WGD event, should have a final set of duplicated genes that have been retained after polyploidisation (Langham et al., 2004; Ming et al., 2008).

To identify the *GGPPS* homologs in *A. thaliana* retained in the *C. papaya* genome, we performed a cross-genome syntenic analysis using the Plant Genome Duplication Database (PGDD, http://chibba.agtec.uga.edu/duplication/). We selected 100 kb of genomic regions adjacent to the *A. thaliana GGPPS* paralogs and the *C. papaya* genome as outgroup. *GGPPS11* and *GGPPS12* are the only paralogs from *A. thaliana*, which have orthologs in syntenic regions of the *C. papaya* genome (Supplementary Figure 3A). Next, we have estimated the relative divergence dates of the *GGPPSs* from *A. thaliana, A. lyrata* and *C. papaya* based on their codon evolution and using an uncorrelated relaxed clock model (see Materials and Methods).

**Figure 4:**
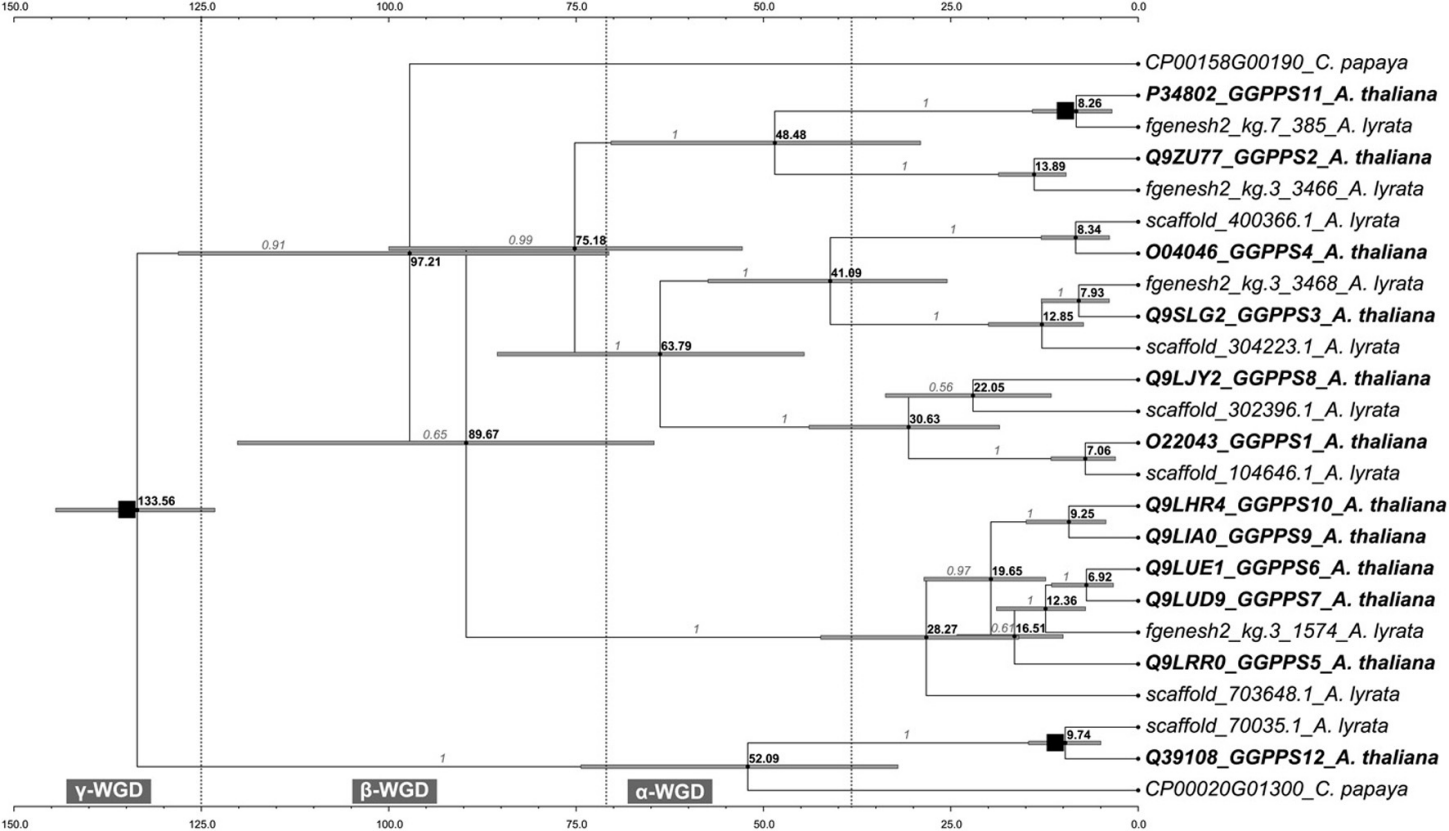
**The calibrated *GGPPS* chronogram**. The maximum clade credibility tree and the estimated divergence dates based on total evidence across 24 homologs from *A.thaliana, A. lyrata* and *C. papaya* are shown. Branch support values are shown in gray. Note the difference in the relative order between the two clades holding GGPPS2, GGPPS11 and GGPPS5-GGPPS7, GGPPS9, GGPPS10 from Figure 1. Both topologies in Figures 1, 4 have high support values but are based on different models of evolution that use amino acid and codon sequences, respectively (see Materials and Methods). Mean divergence dates for all nodes are shown in bold black. Gray bars represent the 95% high posterior density credibility interval for node age. Putative intervals for the WGD events are shown. The most ancient event, common to Arabidopsis, Carica, Vitis, and Populus, is the γ-WGD, which separated monocots and eudicot lineages ca. 125–140 mya (Blanc and Wolfe, 2004; Davies et al., 2004; Jaillon et al., 2007). The following more recent WGDs are assumed to have occurred within the *Brassicales*, with the β event having uncertain position after the point of divergence from *Caricaceae* ca. 72 mya (Ming et al., 2008). The most recent α-WGD that occurred ca. 38–70 mya is placed within the *Brassicaceae* (Bowers et al., 2003; Barker et al., 2009) and predates the divergence of *A. thaliana* and *A. lyrata*, which was estimated to have occurred ca. 13 mya (Beilstein et al., 2010). The nodes used as calibration points are indicated by black squares.

The molecular-dated phylogenetic tree indicates that after the duplication of an ancestral GGPPS within the time range of the oldest γ-WGD one copy evolved into the common ancestor of the extant *GGPPS12* from *A. thaliana* and its orthologs from *A. lyrata* and *C. papaya*. The other copy duplicated ca. 97 mya and evolved into a *GGPPS* gene in *C. papaya* and into the common ancestor of the remaining 11 extant paralogs in *A. thaliana* (*GGPPS1-GGPPS11*) and their orthologs from *A. lyrata* (Figure 4). The *GGPPS* family from the Arabidopsis lineage continued diversifying and expanding during a time range spanning the subsequent β and α-WGD events (Figure 4). As such, during the α-WGD, the extant *GGPPS2* and *GGPPS11* arose (ca. 48 mya) followed by *GGPPS3* and *GGPPS4*, which formed ca. 41 mya (Figure 4). The remaining extant paralogs (*GGPPS1, GGPPS5–GGPPS10*) became fixed in their actual location within the *A. thaliana* genome only after the most recent α-WGD. *GGPPS1* and *GGPPS8* are estimated to have diverged ca. 30 mya, whereas the most recently evolved paralogs in *A. thaliana* are *GGPPS6, GGPPS7, GGPPS9*, and *GGPPS10*, which arose after sequential duplication of their most recent ancestor between 6 and 9 mya (Figure 4).

Generally, following WGD events, many genes return to single copy by fractionation (Lyons et al., 2008). However, some duplicate gene pairs such as genes encoding specialized metabolism enzymes or transcription factors are preferentially maintained (Blanc and Wolfe, 2004; Cannon et al., 2004; Freeling, 2009). Based on the synteny of the surrounding genomic regions, four *GGPPS* paralogs (*GGPPS2, GGPPS3, GGPPS4*, and *GGPPS11*) are found within α-WGD blocks (Bowers et al., 2003; Thomas et al., 2006) (Supplementary Figure 3B). Whereas *GGPPS2* and *GGPPS11* form a pair within one α-WGD block, *GGPPS3* and *GGPPS4* are not retained in pairs with other *GGPPS* paralogs, suggesting that their counterparts were most probably lost due to fractionation processes.

Together, *GGPPS12* appears to be the oldest paralog in *A. thaliana* followed by *GGPPS2-4* and *GGPPS11* (Figure 4). Furthermore, *GGPPS2-4* and *GGPPS11* were found in α-WGD blocks and the dated molecular phylogeny confirms their divergences during the time range of the α-WGD, after the ancestor of Arabidopsis split from *C. papaya*. In contrast to the old paralogs in *A. thaliana, GGPPS6, GGPPS7, GGPPS9*, and *GGPPS10* are paralogs specific to *A. thaliana*. After splitting from *A. lyrata*, the genome of *A. thaliana* experienced a 30% reduction in size and at least nine chromosomal rearrangements (Yogeeswaran et al., 2005; Lysak et al., 2006). Thus, it is possible that the *GGPPSs* specific to *A. thaliana* evolved during these genome reshaping events.

The relative age of the *GGPPSs* corresponds to their divergence in their expression pattern. Old paralogs (e.g., *GGPPS11* and *GGPPS12*) are ubiquitously expressed and at high levels whereas young paralogs (e.g., *GGPPS6* and *GGPPS10*) are predominantly expressed in specific tissues and cell types and generally at lower levels (Figure 3A; Beck et al., 2013) bringing further indication for subfunctionalization of young paralogs.

## Conclusions

The *A. thaliana GGPPS* gene family is an interesting example of gene evolution involving gene duplication followed by neo- and subfunctionalization as well as pseudogenization. GGPPS homologs with canonical protein domain structure are present in all major plant lineages investigated in this study. Nevertheless, it is possible that neofunctionalization of GGPPS paralogs enabled optimized biosynthesis of primary and specialized metabolites. Furthermore, it was recently proposed that functionality inference for the polyprenyl transferases, should not solely rely on primary sequence due to promiscuity of this class of enzymes (Wallrapp et al., 2013). In the case of the GGPPS family from *A. thaliana*, 10 out of 12 predicted isozymes were shown, using *in vitro* and/or *E. coli* complementation assays, to produce GGPP as major product (see Introduction; Zhu et al., 1997a,b; Okada et al., 2000; Wang and Dixon, 2009; Beck et al., 2013). Still, one cannot exclude that some GGPPS will produce longer polyprenyl diphosphates, thereby providing further means of neofunctionalization.

Our functional divergence analysis suggests that changes in the expression patterns of the *GGPPS* paralogs occurring after gene duplication led to developmental and/or condition specific functional evolution. The ancestral states reconstruction showed a highly non-random distribution of developmental expression patterns in the phylogeny, indicating a significant degree of coupling between sequence and developmental expression divergence. This has prompted us to predict that preserving paralogs with different expression may be of importance for the functional divergence of the *GGPPS* paralogs in *A. thaliana.* Moreover, it was recently proposed that the distinct subcellular localization of the GGPPS paralogs may enable a differential allocation of GGPP precursors to downstream isoprenoid pathways, and as such provide an additional mean of their maintenance in the genome (Beck et al., 2013).

The evolutionary pattern of the *GGPPS* gene family in plants, including variation in paralog number mirroring evolution of plant complexity, lineage-specific expansion, neo- and subfunctionalization is consistent with the idea of GGPPSs as flexible enzymes that might have evolved to support adaptation to various specific conditions. This evolutionary pattern can be recognized in many other gene families, in particular those involved in the specialized metabolism: the cytochrome P450-dependent monooxygenases (P450s) (Bak et al., 2011), glucosidases (Kliebenstein et al., 2005) or the terpene synthase family (Tholl, 2006).

It will be interesting to examine by functional analyses of ggpps single and multiple mutants whether the newly evolved *GGPPS* paralogs in *A. thaliana* are functionally redundant or have indeed specific roles in adaptation to various conditions in a distinct spatial-temporal fashion and in response to specific environmental conditions.

### Conflict of interest statement

The authors declare that the research was conducted in the absence of any commercial or financial relationships that could be construed as a potential conflict of interest.

## Supplementary material

Supplementary Figure 1 **Maximum likelihood consensus tree of the GGPPS homologs from plants**. Posterior probabilities are shown. Branch lengths correspond to evolutionary distances. Branch colors represent the major plant lineages: spring green, green algae; orange, mosses; dark green, gymnosperms; and blue, angiosperms.

Supplementary Figure 2 **Amino acid MSA of 119 GGPPS homologs from plants**. The CxxxC motifs are shown in gray. The FARM and SARM motifs are shown in purple.

Supplementary Figure 3 **Syntenic relationships of *GGPPS* paralogs from *A. thaliana* using *C. papaya* as outgroup. (A)** Blocks duplicated by WGD and harboring *GGPPS11* and *GGPPS12* are shown. Their orthologs found in syntenic region of *C. papaya* genome are indicated by red connecting lines. **(B)** *GGPPS2, GGPPS3, GGPPS4* and *GGPPS11* paralogs from *A. thaliana* found within α-WGD blocks on chromosome 2 and 4, respectively, are shown. Only *GGPPS2* and *GGPPS11* are retained as a pair (connected by red line), whereas the counterparts of *GGPPS3* and *GGPPS4* appear to have been lost from the corresponding syntenic region. Each genomic region spans 100 kb. The *GGPPS* paralogs and their orthologs from *C. papaya* are shown as red arrows. Blue arrows indicate anchor genes and they are connected by blue lines if retain within a WGD block.

Supplementary Table 1 **119 GGPPS protein sequences used for the phylogenetic tree reconstruction**.

Supplementary Table 2 **Polyprenyl synthase domain evolution**.

Supplementary Dataset 1 **MAFFT MSA in FASTA format of 119 GGPPS homologs from plants**.
