# Supplementary material for: Distinct evolutionary strategies in the GGPPS family from plants
Source: Front Plant Sci. 2014 May 27;5:230. doi: 10.3389/fpls.2014.00230 (PMC4034038; doi:10.3389/fpls.2014.00230)
Supplement: Supplementary Figure 1 — Maximum likelihood consensus tree of the GGPPS homologs from plants. Posterior probabilities are shown. Branch lengths correspond to evolutionary distances. Branch colors represent the major plant lineages: spring green, green algae; orange, mosses; dark green, gymnosperms; and blue, angiosperms. [file DataSheet1.ZIP › SupplementaryFigure1.pdf]

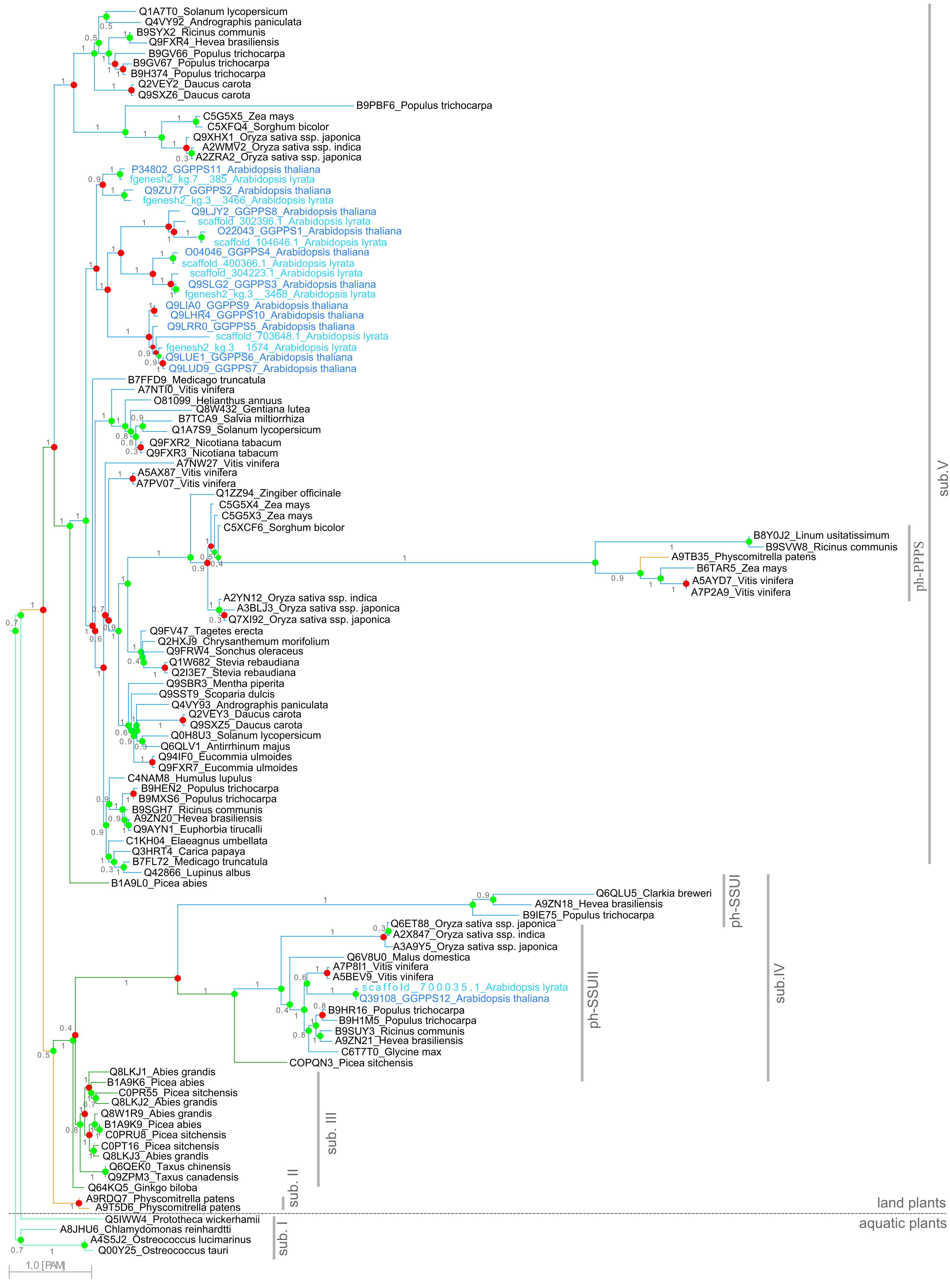

Figure S1. Maximum likelihood consensus tree of the GGPPS homologs from plants. Posterior probabilitites are shown. Branch lengths correspond to evolutionary distances. Branch colors represent the major plant lineages: spring green-green algae, orange-mosses, dark green-gymnosperms and blue-angiosperms.
