# Supplementary material for: Distinct evolutionary strategies in the GGPPS family from plants
Source: Front Plant Sci. 2014 May 27;5:230. doi: 10.3389/fpls.2014.00230 (PMC4034038; doi:10.3389/fpls.2014.00230)
Supplement: Supplementary Figure 1 — Maximum likelihood consensus tree of the GGPPS homologs from plants. Posterior probabilities are shown. Branch lengths correspond to evolutionary distances. Branch colors represent the major plant lineages: spring green, green algae; orange, mosses; dark green, gymnosperms; and blue, angiosperms. [file DataSheet1.ZIP › SupplementaryFigure3.pdf]

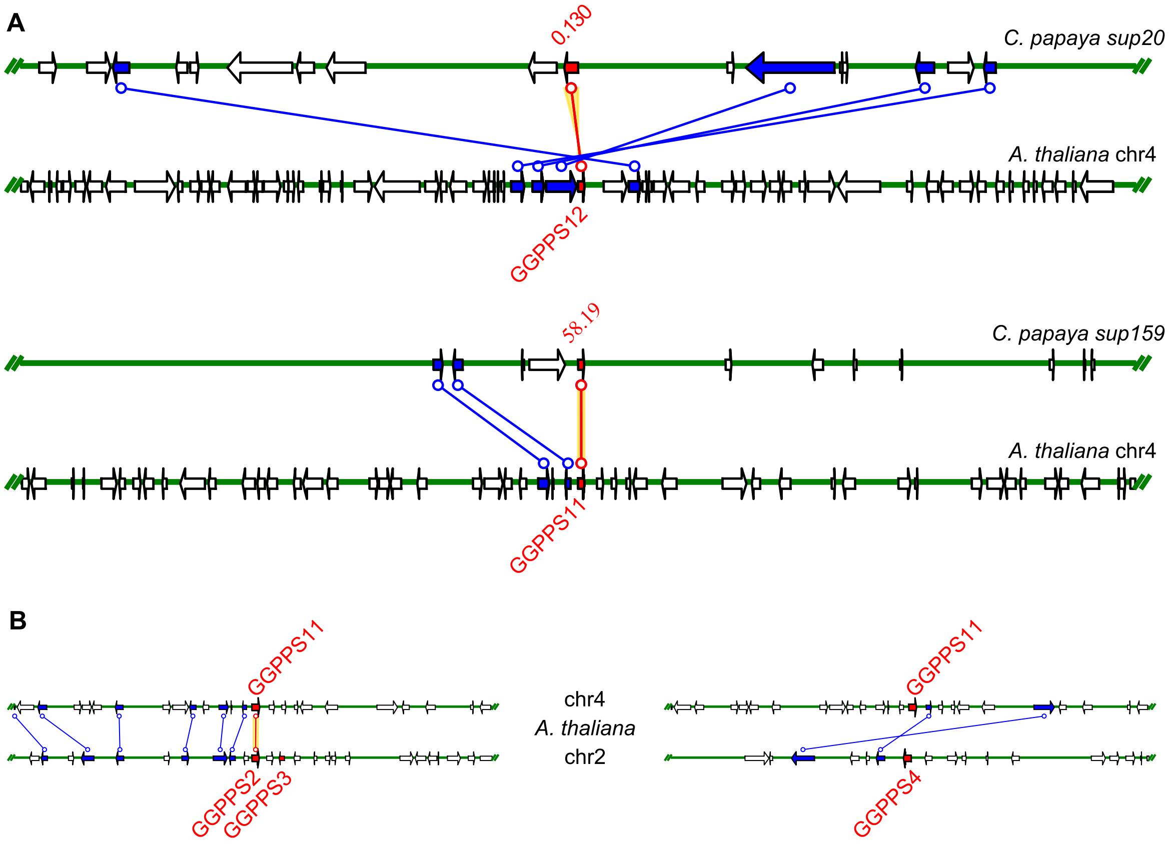


**Figure S3.** Syntenic relationships of the *GGPPS* paralogs from *A. thaliana* using *C. papaya* as outgroup. **A.** Blocks duplicated by WGD and harbouring *GGPPS11* and *GGPPS12* are shown. Their orthologs found in syntenic region of *C. papaya* genome are indicated by red connecting lines. **B.** *GGPPS2, GGPPS3, GGPPS4* and *GGPPS11* paralogs from *A. thaliana* found within α-WGD blocks on chromosome 2 and 4, respectively, are shown. Only *GGPPS2* and *GGPPS11* are retained as a pair (connected by red line), whereas the counterparts of *GGPPS3* and *GGPPS4* appear to have been lost from the corresponding syntenic region. Each genomic region spans 100 kb. *GGPPS* paralogs and their orthologs from *C. papaya* are shown as red arrows. Blue arrows indicate anchor genes and they are connected by blue lines if retained within a WGD block.
